# Highly efficient capture of cancer cells expressing EGFR by microfluidic methods based on antigen-antibody association

**DOI:** 10.1038/s41598-018-30511-9

**Published:** 2018-08-13

**Authors:** Takashi Ohnaga, Yoshinori Takei, Takuya Nagata, Yutaka Shimada

**Affiliations:** 1 Central Research Laboratories, Toyama Industrial Technology Center, 150 Futagami-cho, Takaoka, Toyama 933-0981 Japan; 2 Department of Nanobio Drug Discovery, Graduate School of Pharmaceutical Sciences, Kyoto University, 46-29 Yoshida Shimoadachi-cho, Sakyo-ku, Kyoto 606-8501 Japan; 3 Department of Surgery and Science, Graduate School of Medicine and Pharmaceutical Sciences, University of Toyama, 2630, Sugitani, Toyama 930-0194 Japan

## Abstract

Epidermal growth factor receptor (EGFR) was evaluated as a target antigen for cancer cell capture by microfluidic methods based on antigen-antibody association. A polymer CTC-chip microfluidic device was surface-functionalized with three different anti-EGFR antibodies and used to capture EGFR-expressing cancer cells. Capture efficacy depended on the type of antibody used, and cetuximab efficiently captured cancer cell lines that had a wide range of EGFR expression. Capture efficiency was analyzed from the viewpoint of antigen-antibody association in a kinetic process, i.e., cell rolling well-known in leukocyte adhesion, and antibodies with a smaller dissociation constant were shown to result in more efficient capture. Moreover, a lower limit of cellular EGFR expression level for the capture was estimated and methods to decrease the limit were discussed based on densities of anti-EGFR antibody on the device surface.

## Introduction

Immune-based capture of cells is commonly used for cell screening and has been applied to isolation of cancer cells that detach from solid tumors and disseminate into the peripheral blood of patients. These cells, known as circulating tumor cells (CTCs), are attractive for cancer diagnosis, therapy and research but difficult to isolate because of extreme rarity in patient blood^1,2^. Although conventional immune-based capture of CTCs relies on immunomagnetic enrichment, recent advances in microfluidic technologies have allowed improving CTC isolation methods^3–5^. Because immune-based capture depends on the molecular interaction between cell surface antigens and antibodies, frequent contact between the target cell and antibody-immobilized surface is needed for highly efficient capture. Microfluidic devices achieve this requirement because of an enhanced surface-to-volume ratio of microstructures^6^. The so-called ‘CTC-chip’ with surface microstructures comprised of several tens of thousands of microposts covered with antibody captured CTCs successfully from patients with various cancer types in clinical tests^7^, which gave rise to the worldwide development of this kind of microfluidic devices^8^.

However, these devices could not always detect CTCs, and this is partly because they mostly used antibodies only against epithelial cell adhesion molecule (EpCAM). Because EpCAM is expressed exclusively in epithelia and epithelial-derived neoplasms, anti-EpCAM antibody is widely applied to immune-based capture of cancer cells in blood so far. However, EpCAM expression varies among cancer cells and is upregulated or downregulated in response to an external stimulus^9^. It is well known that downregulation of EpCAM by epithelial mesenchymal transition (EMT) leads to the failure in CTC detection by EpCAM-based techniques^10,11^. We developed another type of CTC-chip device, called ‘polymer CTC-chip’^12^. The chip produced with UV light-curing resins is transparent to visible and UV light and mechanically tough compared to conventional silicon chips, and can be commercially provided at low cost. Moreover, since the resin contains functional groups which react with proteins just by contacting them and has lasting surface reactivity, antibodies can be selected by chip-users arbitrarily at any time and immobilized onto chip easily. We have reported both EpCAM-dependent and -independent capture of cancer cells using the polymer CTC-chip^12–15^.

In this study, we applied this polymer CTC chip to capture of cancer cells expressing epidermal growth factor receptor (EGFR). EGFR is a 170 kDa transmembrane protein with intrinsic tyrosine kinase activity that regulates cell growth and is overexpressed in many cancers^16^. Moreover, because EGFR expression is reported to increase in tumor cells undergoing EMT^10^, EGFR seems attractive as a target for CTC capture and to contribute to CTC detection. We investigated different anti-EGFR antibodies and levels of EGFR expression of cancer cells on capture performance in order to establish capture conditions for clinical applications. Mesenchymal-like cells expressing EGFR were included in the investigation.

We were particularly interested in influence of antigen-antibody association on the cell capture by microfluidic methods here. Among factors which affect immune-based capture with microfluidic devices, frequent contact between cell and device surface is important. Therefore, design of microstructures has been often discussed and appropriate microstructures for efficient capture of CTC have been known in the microfluidic devices such as CTC-chip, HB-chip^17^, GEDI-chip^18^ and GEM-chip^19^. In contrast, even though cell adhesion to device surface has a major influence on this cell capture, understanding of antigen-antibody association in the capture seemed inadequate. We analyzed capture efficiency from the viewpoint of antigen-antibody association at equilibrium and in a kinetic process. In addition, because formation of antigen-antibody complexes depends on concentrations of these components, influence of surface density of anti-EGFR antibody was also discussed.

## Results

### Capture of cancer cells expressing EGFR with different antibodies

**Figure 1:**
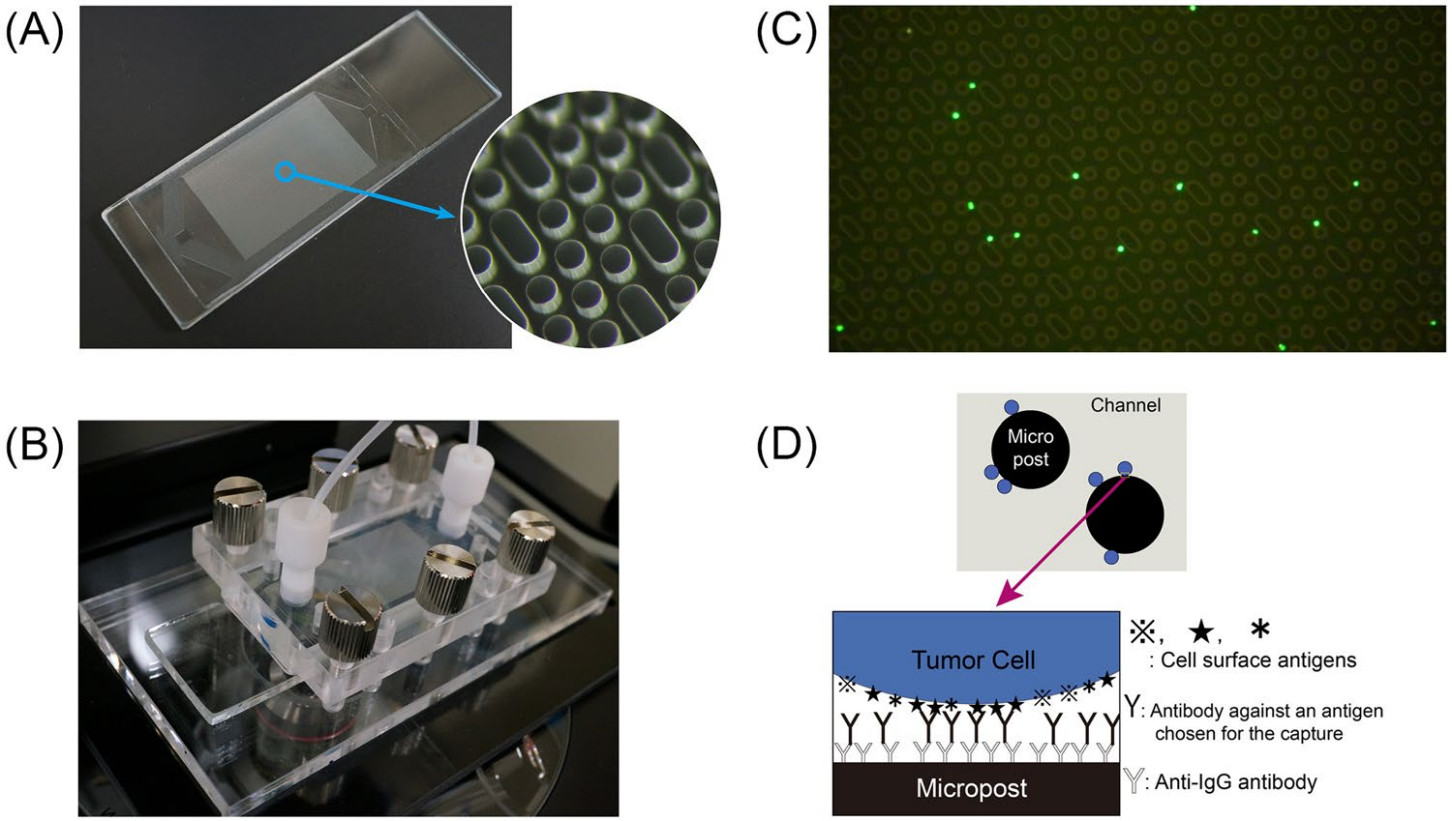
**(A)** The polymer CTC-chip has a size of 75 mm × 25 mm. The diameter and height of the posts in the magnified image are 100 µm. **(B)** The chip set in a holder enables a liquid sample to flow through the channel. The two ports of the holder were connected to a syringe pump and a sample tube with tubing and fittings. **(C)** Fluorescent image of KYSE220 cells caught on the chip surface immobilized with cetuximab. The cells are labeled fluorescently before the capture test. **(D)** A scheme to capture cancer cells with the polymer CTC-chip. The antibody for capture is selectable.

**Figure 2:**
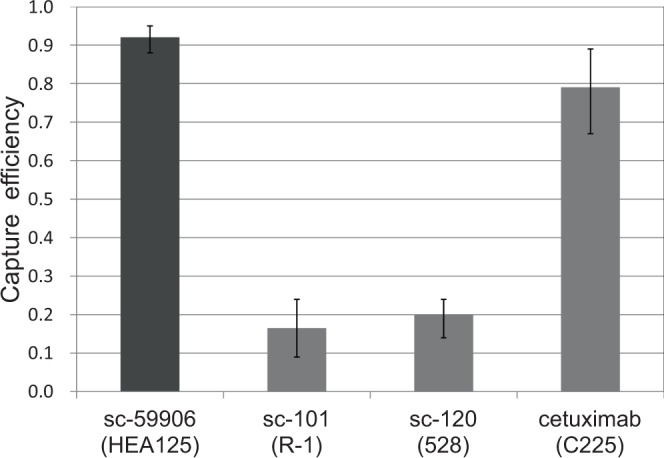
Capture efficiencies for the esophageal cancer cell line KYSE220 obtained through the capture tests using anti-EGFR antibodies sc-101, sc-120 and cetuximab, and anti-EpCAM antibody sc-59906^13^.

The polymer CTC-chip (Fig. 1A) was set in a holder that enabled delivery of samples (Fig. 1B) to capture tumor cells from the esophageal cancer cell line KYSE220. Fluorescently labeled cells were successfully caught on the chip surface immobilized with cetuximab (Fig. 1C) according to the scheme shown in Fig. 1D. We measured the number of caught cells (N_c_) compared to the number of cells supplied to the chip (N_f_) to calculate a capture efficiency defined as N_c_/N_f_. Three different anti-EGFR antibodies, sc-101, sc-120 and cetuximab were immobilized on different chip surfaces, and the capture efficiencies for each are shown in Fig. 2 in comparison to that for anti-EpCAM antibody sc-59906 reported previously^13^. The capture efficiencies were also estimated by use of breast cancer cell line expressing low-EGFR, MCF-7^20^ as controls and were 0.08 for sc-120 and 0.07 for cetuximab. Capture efficiency depended on the type of immobilized anti-EGFR antibody, and cetuximab had the highest capture efficiency, which was similar to that for anti-EpCAM antibody.

### Analysis of antibody binding to cell

**Figure 3:**
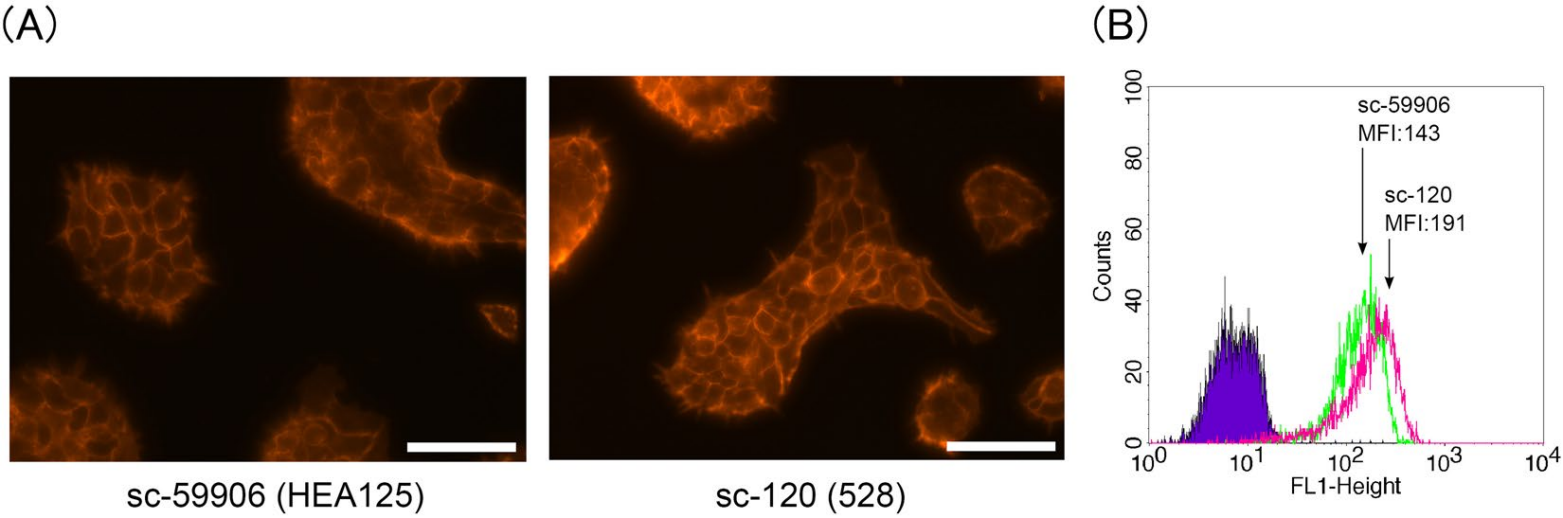
**(A)** Fluorescent images of KYSE220 cells stained with sc-120 or sc-59906, and Cy3 anti-mouse IgG antibody. The scale bar indicates 100 μm. **(B)** Flow cytometry analysis of KYSE220 cells stained with sc-120 or sc-59906, and Alexa Fluor 488-labeled anti-mouse IgG antibody. The x-axis shows logarithmic fluorescence intensity. Mean fluorescence intensity (MFI) was calculated from the flow cytometry data.

KYSE220 cells were immunofluorescently stained with antibodies used for the capture test and analyzed by microscopic observation and flow cytometry. To keep the affinity between primary and secondary antibodies constant, mouse antibodies sc-120 and sc-59906, and a common fluorescent secondary antibody were used. In the microscopic observation, fluorescence intensities of the stained cells were not so different for these two antibodies or slightly higher for sc-120 than for sc-59906 (Fig. 3A). The flow cytometry analysis showed that mean fluorescence intensity (MFI) of the cell stained with sc-120 (MFI: 191) was higher than that stained with sc-59906 (MFI: 143) (Fig. 3B). These results indicated that the number of antibody molecules binding to a single KYSE220 cell was higher for sc-120 than for sc-59906.

### Capture of cancer cells exhibiting wide range of EGFR expression

**Figure 4:**
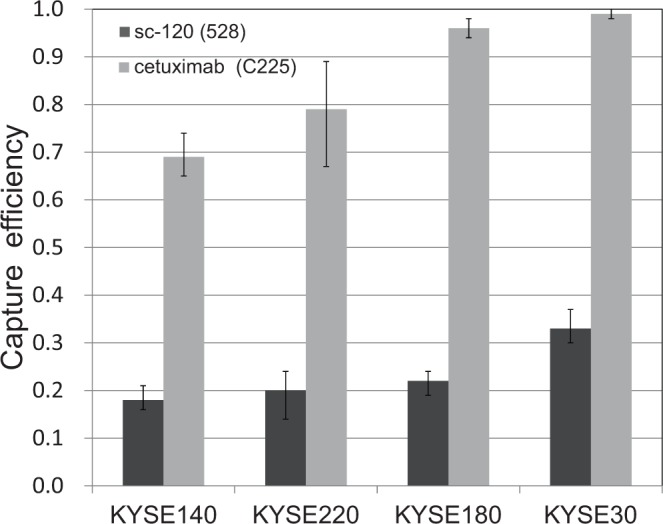
Capture efficiencies for esophageal cancer cell lines KYSE140, KYSE220, KYSE180 and KYSE30 obtained through the capture tests using anti-EGFR antibodies sc-120 and cetuximab.

We performed the capture tests using esophageal cancer cell lines KYSE140, KYSE180 and KYSE30 in addition to KYSE220. These cell lines exhibit a wide range of EGFR expression from 6.0 × 10^4^ to 1.2 × 10^7^ receptors/cell^21^. Capture efficiencies were evaluated with sc-120 and cetuximab antibodies (Fig. 4). For cetuximab, most cells were captured at the highest EGFR expression level (in KYSE30). Capture efficiency decreased with reduced EGFR expression levels but remained relatively high at the lowest level of EGFR expression (in KYSE140). For sc-120, capture efficiency was low for all cell lines but increased slightly with increased expression of EGFR.

### Capture of mesenchymal-like cancer cells

**Figure 5:**
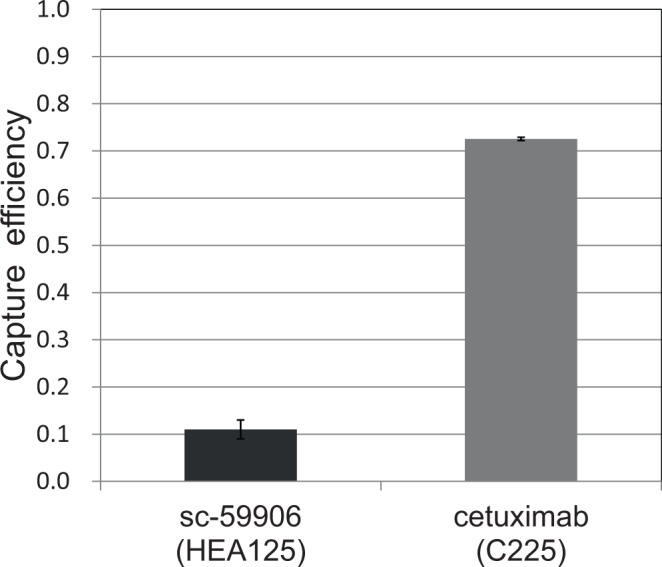
Capture efficiencies for the breast cancer cell line MDA-MB-231 obtained through the capture tests using cetuximab and sc-59906^13^.

The breast cancer cell line MDA-MB-231 that expresses a low level of EpCAM^22^ was used for the capture with cetuximab (Fig. 5). The obtained capture efficiency was much higher than that for the anti-EpCAM antibody sc-59906 reported previously^13^.

## Discussion

For immune-based capture of cancer cells based on microfluidic methods, EGFR has been used as a target marker in a few studies^23,24^. However, since these studies were performed with antibody mixtures containing anti-EGFR antibody, the capture behavior only by anti-EGFR antibody has not been understood so far. Here, we elucidated applicability of EGFR for the capture using the polymer CTC-chip. Because the capture was affected by a type of antibody, we investigated the relationship between antibody binding to cell and the capture efficiency for different antibodies. Analysis of immunofluorescently stained cells of KYSE220 with antibody sc-120 or sc-59906 indicated that the number of antibody molecules binding to a single KYSE220 cell was higher for sc-120 than for sc-59906 (Fig. 3). However, in the capture of KYSE220 cells by the polymer CTC-chip, there was a large difference in capture efficiencies for sc-120 (0.2) and sc-59906 (0.92), and antibody binding to cell was more suppressed for sc-120 than for sc-59906. We think this suppression is caused partly by immobilization of antibody onto the chip surface^25^, which reduces the mobility of molecules and reactivity to antigen. Moreover, the suppression may be attributed to other factors, such as motion energy of flowing cells, which must be dissipated for cell adhesion, and formation of antigen-antibody complexes on short time scales. Decrease in capture efficiency caused by elevated sample flow rate^7,17,19^, which increases the motion energy and shortens a time scale of the complex formation, may result from these factors.

Thus, cell capture on the microfluidic device surface is a kinetic process, and therefore we examined cell motion during capture to further investigate difference in capture efficiency. We observed that cells collided with microposts in the chip, rolled and then stopped (see Supplementary Video S1), which resembled the cell rolling and adhesion of leukocyte on vascular surfaces in the body^26^. Leukocyte adhesion under these conditions depends on the association and dissociation rates of ligands and receptors^26,27^. For initial adhesion of circulating cells, fast association is necessary, which is related to a larger k_on_, where k_on_ is the association rate constant in a reaction between ligand and receptor. Once a tether is formed, slow dissociation with a smaller k_off_, the dissociation rate constant, regulates rolling velocities and causes cell adhesion. Thus, these rate constants partly explain cell adhesion for rolling cells and predict a smaller dissociation constant K_d_ expressed as k_off_/k_on_ at equilibrium for the firm adhesion. The dissociation constants for clone 528 antibody and cetuximab are known from anti-cancer studies^28^. These antibodies were produced for anti-cancer treatments to prevent EGFR function by binding to the receptor. Cetuximab has a small K_d_ = 0.39 nM^28,29^ compared with that of the 528 antibody K_d_ = 1.5–2.0 nM^28^. Because a smaller K_d_ results in tighter cell adhesion, our findings that antibodies with smaller K_d_ were more favorable for efficient capture by the polymer CTC-chip agree with these studies. However, further capture tests with antibodies exhibiting a much smaller K_d_, such as panitumumab (K_d_ = 0.05 nM)^30^, are needed because there have been cases where adhesion occurred under conditions of a larger k_off_^27^, which suggests that we do not fully understand immune-based capture of cancer cells by immobilized antibodies.

For clinical use, the lower limit of cellular EGFR expression for efficient capture by the polymer CTC-chip had been unclear. In this study, the chips using cetuximab could sufficiently recover the cancer cells with an EGFR expression level more than that of KYSE140 (6.0 × 10^4^ receptors/cell). As for other esophageal cancer cell lines, most cells have higher EGFR expression levels than that of KYSE140^21^, which suggests that this chip may be used to isolate CTCs from many types of esophageal cancer. However, capture efficiency was reduced by low levels of EGFR expression, and the CTC-chip is likely insufficient to capture cancer cells with much lower EGFR expression. In fact, there are some breast cancer cell lines that have expression levels of EGFR lower than that of KYSE140^20^. In Fig. 4, despite of a large difference in EGFR expression levels, capture efficiencies did not markedly differ for sc-120 and cetuximab. In other case of cell binding to a plane covered by a ligand where ligand density exceeded cellular receptor density, adhesion depended weakly on ligand density, but strongly on receptor density^31^. This implies that the surface densities of anti-EGFR antibody were not high compared to those of surface EGFR in esophageal cancer cell lines and association saturated in this study. Therefore, increasing antibody density may improve chip performance. In the reaction between antibody and functional groups on the chip surface, higher concentration of antibody simply elevates antibody density on the surface, but may increase costs and reduce chip availability for clinical use. Increase of the number of functional groups is another option to enhance the antibody density and can be attained by use of polyfunctional macromolecules such as dendrimers^32^. So many functional groups in the macromolecule immobilized on the chip surface are distributed three-dimensionally and can promote the reaction with antibody.

MDA-MB-231 cells were captured by the polymer CTC-chip using cetuximab due to high EGFR expression, which is more than 10^5^ receptors/cell^11,20,33^. Because this expression level is comparable to that of KYSE220 (1.3 × 10^5^ receptors/cell), the capture efficiencies for these cell lines were similar. Thus, this chip is useful for the capture of mesenchymal-like cells, even though a range of cancer type which anti-EGFR antibody can capture may be narrower than that anti-EpCAM antibody captures.

## Methods

### Cell lines and antibodies

The human esophageal cancer cell lines, KYSE40, KYSE220, KYSE180, and KYSE30 were provided by Dr. Yutaka Shimada^34^ and have an EGFR expression of 6.0 × 10^4^, 1.3 × 10^5^, 6.0 × 10^5^, and 1.2 × 10^7^ receptors/cell, respectively^21^. The breast cancer cell lines, MCF-7 and MDA-MD-231 were obtained from ATCC (Manassa, VA, USA). Mouse anti-human EGFR antibodies sc-101 (clone R-1) and sc-120 (clone 528) and mouse anti-human EpCAM antibody sc-59906 (clone HEA125) were purchased from Santa Cruz Biotechnology, Inc. (Santa Cruz, CA, USA). Cetuximab (chimeric version of anti-EGFR antibody of clone 225), also known as Erbitux, was obtained from Bristol-Myers Squibb (New York, NY, USA). Goat anti-mouse IgG antibody 1032–01 and rabbit anti-human IgG antibody 6005–1 were purchased from Southern Biotech (Birmingham, AL, USA). Cyanine3 (Cy3) goat anti-mouse IgG antibody CLCC35010 was purchased from Cedarlane (Hornby, ON, Canada). Alexa Fluor 488-labeled donkey anti-mouse IgG antibody A-21202 was purchased from Thermo Fisher Scientific, Inc. (Waltham, MA, USA).

### Cell culture and analysis of immunofluorescently stained cells

All cell lines were grown at 37 °C and 5% CO_2_ with humidity. Esophageal cell lines and MCF-7 were cultured in Dulbecco’s modified eagle medium (Gibco, Invitrogen, Carlsbad, CA, USA) containing 4.5 g/L L-glutamine supplemented with 10% fetal bovine serum and 1% penicillin-streptomycin (Gibco). MDA-MB-231 was cultured in Leibovitz’s L-15 Medium (Wako Pure Chemical Industries Ltd., Japan) containing L-glutamine supplemented with 15% fetal bovine serum and 1% penicillin-streptomycin (Gibco).

For microscopic observation of immunofluorescently stained cells, KYSE220 cells cultured in 96-well microplates were fixed in 4% paraformaldehyde (PFA) (Wako Pure Chemical Industries) for 30 min and washed with phosphate buffered solution (PBS). Mouse antibody of sc-59906 or sc-120 at a concentration of 20 µg/mL was applied to the cells for 1 h at room temperature. The cells were washed with PBS and stained with Cy3 anti-mouse IgG antibody CLCC35010 at a concentration of 4 µg/mL for 30 min. Following a final wash with PBS, fluorescent images were captured by an inverted microscope CKX41 (Olympus, Japan) equipped with a Peltier-cooled CCD camera CL500 (Wraymer, Japan).

For analysis by flow cytometry, KYSE220 cells were harvested with Cell Dissociation Solution (Sigma-Aldrich, St. Louis, MO, USA) and resuspended in PBS at 5 × 10^5^ cells/mL. The cells were fixed with 4% PFA for 10 min at 4 °C, and washed twice with PBS. The fixed cells were incubated with either sc-120 antibody or sc-59906 antibody at a concentration of 20 µg/mL at 4 °C. As a negative control, cells were incubated without antibody. After incubation for 1 hour, cells were washed by centrifugation at 1000 × g for 5 min at 4 °C. The cells were resuspended in PBS containing Alexa Fluor 488-labeled anti-mouse IgG antibody at a concentration of 4 µg/mL for 30 min at 4 °C. The cells were analyzed by BD FACSCalibur (BD Biosciences, San Jose, CA, USA).

### Chip fabrication and antibody immobilization

Fabrication of the polymer CTC-chip was performed as described previously^12^. Briefly, UV light-curable resin with epoxy functional groups was poured into a mold and covered with a glass plate, and then the resin was cured by exposure to UV-light through the glass plate for 3 min. After the cure reaction was complete, the mold was released. The microstructure pattern of the chip was designed on the basis of the previous reports. Briefly, repeated patterns of equilateral triangular arrays of microposts with a height and a diameter of 100 µm were modified to maximize the interactions between microposts and cells^7^. Since cells were trapped physically in the gaps between two posts located close together, we redesigned the microstructure and removed the gap by joining two posts together^12^. The mold was made by the fabrication of a silicon wafer using deep reactive ion etching. Antibody immobilization of the chip was performed as follows. The chip surface of the resin was covered with antibody solution of anti-mouse IgG antibody 1032–01 or anti-human IgG antibody 6005–1 in PBS at a concentration of 20 µg/mL overnight and then washed. Next, anti-human EGFR antibody sc-101, sc-120 or cetuximab in PBS at a concentration of 20 µg/mL was allowed to react with the corresponding IgG antibody on the chip for 1 h before a wash with PBS. The chip surface was kept wet until use.

### System for cell capture and observation

The system for cell capture and observation was described previously^12^. Briefly, the polymer CTC-chip immobilized with surface antibody was set in a holder (Fig. 1B), which enabled a liquid sample to flow through the channel, and then the two ports of the holder were connected to a syringe pump and a sample tube with tubing and fittings. The holder was placed on a sample stage of CKX41 equipped with a digital video camera, HDR-CX590V (Sony, Japan), and then images and movies of cells in the chip were recorded.

### Cell capture test

Cell capture tests using the above system were performed as described previously^12^. Cells grown in culture dishes were collected with trypsin-ethylene diamine tetra acetic acid (Wako Pure Chemical Industries) and fluorescently labeled with CellTrace (Life Technologies, Rockville, MD, USA) using the manufacturer’s protocol. Cells were suspended in PBS containing 1% bovine serum albumin (Wako Pure Chemical Industries) at a concentration of 300 cells/mL. The cell suspension sample was put in the sample tube of the cell capture system and sent to the chip using the syringe pump at a volumetric flow rate of 1.5 mL/h for 1 h. The sample tube was shaken to prevent precipitation and cell adhesion. After the sample supply was completed, the channel of the chip was washed with PBS. The capture efficiency was determined from the number of caught cells in the chip (N_c_) and that of cells supplied to the chip (N_f_). We took images of the whole chip with HDR-CX590V and counted cells for N_c_. N_f_ was measured as the number of cells that flowed through the chip inlet in the movie recorded during the cell capture test with HDR-CX590V.

## Electronic supplementary material

Supplementary Information

Supplementary Video S1
