# Supplementary material for: Highly efficient capture of cancer cells expressing EGFR by microfluidic methods based on antigen-antibody association
Source: Sci Rep. 2018 Aug 13;8:12005. doi: 10.1038/s41598-018-30511-9 (PMC6089922; doi:10.1038/s41598-018-30511-9)
Supplement: Supplementary file 1 — Supplementary Information [file 41598_2018_30511_MOESM1_ESM.pdf]

Supplementary Information

Highly efficient capture of cancer cells expressing EGFR by microfluidic methods based on antigen-antibody association

Takashi Ohnaga[a,*], Yoshinori Takei[b] , Takuya Nagata[c] and Yutaka Shimada[b]

[a] Central Research Laboratories, Toyama Industrial Technology Center, 150 Futagami-cho, Takaoka, Toyama 933-0981, Japan

[b] Department of Nanobio Drug Discovery, Graduate School of Pharmaceutical Sciences, Kyoto University, 46-29 Yoshida Shimoadachi-cho, Sakyo-ku, Kyoto 606-8501, Japan

[c] Department of Surgery and Science, Graduate School of Medicine and Pharmaceutical Sciences, University of Toyama, 2630 Sugitani, Toyama 930-0194, Japan

[*] Corresponding author: Takashi Ohnaga, Toyama Industrial Technology Center, 150 Futagami-cho, Takaoka, Toyama 933-0981, Japan; Phone: +81-766-21-2121, Fax: +81-766-21-2402; E-mail: ohnaga@itc.pref.toyama.jp

Supplementary Video S1 Motion of KYSE220 cells in the polymer CTC-chip immobilized with cetuximab during the capture test. Cells were suspended in PBS containing 1% bovine serum albumin at a concentration of 2000 cells/mL. The cell suspension sample was sent to the chip at a volumetric flow rate of 1.5 mL/h.
